# Health Impacts of Climate Change-Induced Subzero Temperature Fires

**DOI:** 10.3390/ijerph14070814

**Published:** 2017-07-20

**Authors:** Maria-Monika Metallinou, Torgrim Log

**Affiliations:** Department of Engineering, Western Norway University of Applied Sciences, 5528 Haugesund, Norway; Torgrim.Log@hvl.no

**Keywords:** cold climate fires, health impacts, risk identification, risk management

## Abstract

General fire risk and the special risk related to cold climate cellulosic drying processes are outlined. Four recent subzero temperatures fires are studied with respect to health impacts: a wooden village fire, a single wood structure fire, a wildland urban interface (WUI) fire and a huge wildland fire. The health impacts range from stress related to loss of jobs, psychological effects of lost possessions, exposure to smoke and heat as well as immediate, or delayed, loss of lives. These four fires resulted in 32 fatalities, 385 persons hospitalized for shorter or longer periods, 104 structures lost and 1015 km^2^ of wildland burned north of, and just south of, the Arctic Circle. It is shown that the combination of subzero temperature dry weather, strong winds, changing agricultural activities and declining snowpack may lead to previously anticipated threats to people and the environment. There are reasons to believe that these fires are a result of the ongoing climate changes. Risk impacts are discussed. Rural districts and/or vulnerable populations seem to be most affected. Training methods to identify and better monitor critical fire risk parameters are suggested to mitigate the health impacts of a possibly increasing number of such fires.

## 1. Introduction

It is well known that fires represent a threat to people. In previous centuries it was considered the most severe threat to wooden villages and towns during peace time and most towns experienced at least one devastating conflagration (major fire) during their history. Some towns were regularly haunted by huge fires, often caused by hot work. As a risk-reducing measure, hot work could be banned and moved out of the town. A well-known example is the city of Venice, Italy, where the glassblowing industry was forced to move to the island of Murano in the XIIIth century to limit the fire risk.

Most people associate fires with dry and warm weather. Major conflagrations have, however, also been experienced during the winter time. The most severe conflagration in modern times in Norway, i.e., the Ålesund conflagration, 23 January 1904, may serve as an example. That fire became out of control in storm strength wind (22–30 m/s wind speed), killed one person, destroyed 850 out of 1080 structures and resulted in 10,000–12,000 homeless people [1].

During the 20th century fewer conflagrations were observed. This may partly be due to more efficient firefighting equipment, and awareness of the fire risk, etc. This fire risk is, however, not always under control and the list of major conflagrations is still long. As an example, Sakata (Japan), experienced a massive conflagration on 29 October 1976 [2]. Despite an efficient system of fire hydrants and portable fire extinguishing systems operated by the civilians, they had to withdraw due to the untenable conditions and leave the firefighting to the professionals. The firefighters were, however, only able to prevent the fire from spreading sideways and did not get it under control before it reached the Niidagawa River after destroying 1744 buildings [3].

Wildland Urban Interface (WUI) fires are, however, an increasing risk issue as an increasing number of structures are lost every year, in particular in the USA, the Mediterranean area and in Australia. The Witch and Guejito fires in October 2007, in the Trails community (San Diego, CA, USA) that destroyed 74 residences may serve as an example [4]. When establishing new boomtowns in previously scarcely populated areas, and without proper risk assessment and risk mitigation, major incidences like the devastating Canadian Fort McMurray fire, 1 May to 5 July 2016, may occur [5]. During three weeks the fire burned 3000 km² and forced the evacuation of 88,000 residents. It destroyed thousands of homes and buildings and was expected to negatively affect the Canadian gross domestic product (GDP). It is likely that this fire was related to climate changes as well as poor understanding of the immanent risk by the decision makers [5].

During the last few years several unusual and severe cold climate fires have occurred. Four of these recent subzero temperature fires will be analyzed with respect to human health impacts. These are: a 1000 km^2^ wildfire in the Alaskan tundra North Slope, north of the Arctic Circle [6], a Wildland Urbane Interface (WUI) heather fire in Norway, just south of the Arctic Circle [7], a village fire in Norway [8] and a nursing home fire in Quebec, Canada [9]. Before analyzing these fires, the general dangers of fires to humans, i.e., civilians and firefighters, as well as the impact of megafires on the environment are explained. In view of the analysis of each separate fire, and external parameters influencing the fire risk, some general conclusions regarding the possible ways forward are drawn.

## 2. The Dangers of Fire to the Human Health

### 2.1. Heat Release Rate (HRR) in Fires

An accidental fire is an uncontrolled combustion process. The most common fuel is wood, or similar cellulose based materials, which mostly consist of carbon, oxygen and hydrogen. Wood may be chemically approximated by the formula CH_1.5_O_0.75_ [10]. The corresponding stoichiometric reaction with oxygen is:

CH_1.5_O_0.75_ + O_2_ → CO_2_ + 0.75 H_2_O + heat (1)

During wood pyrolysis a number of different complex products are formed, most of which end up as invisible gases or visible smoke products. Some of these are carcinogenic and some, such as acrolein and HCN, are poisonous. Regardless of minor quantities of these products all fires produce CO, which generally is responsible for most fire fatalities [11].

The flame temperatures are typically about 1000 °C. Inhalation of hot gases at 1000 °F (538 °C) will immediately result in heat injury to the upper airways above the carina [12]. Another major problem is the risk of skin burns. Skin injury starts to develop when the skin temperature is greater than 44 °C as a result of protein breakdown [13,14]. The skin damage is a function of temperature and time above this threshold temperature and the damage develops exponentially with excess temperature [15]. Flames therefore represent a significant threat also for very short exposure periods [16]. In 2004 fires resulted in 11 million burn injuries hospitalizations [17] and in high income countries there were 0.14 burn injuries hospitalizations per 100,000 persons [18].

Firefighters close to the fire as well as people far away are also exposed to Particulate Matter (PM). This is evidenced by increased demand for asthma medicine as well as lower birth weights in the wildfire season [19,20,21].

Wildland megafires represent an indirect threat to people’s health due to the huge amounts of CO_2_ (greenhouse gas) being released to the atmosphere (Equation (1)). The soil may also become involved in the combustion processes, not only in warmer climates, but also in temperate areas [22]. During a Scottish Highlands fire in 2006, an organic soil loss of about 773 t, corresponding to about 396 t C, i.e., 1 450 t CO_2_ to the atmosphere was registered. Globally, more carbon is stored in peat land than in vegetation, and the amount is similar to that in the atmosphere. Peat megafires are the largest fires on Earth with respect to fuel consumption and can consume 100 times more fuel per unit area than flaming fires [23]. Wildfires may also contribute to global warming after the fire is extinguished by altering the soil heat absorption, evidenced by deeper thaw depths in permafrost due to reduced albedo during the early stages of succession [24].

### 2.2. Heat Release Rate (HRR) in Fires

The Heat Release Rate (HRR) is the key parameter characterizing fire behavior [25]:

(2) $${\dot{Q}}_{c}=A_{f}\cdot {\dot{m}}_{f}^{"}\cdot \chi \cdot ∆H_{c}\;(W)$$

where $A_{f}$ (m^2^) is the surface area of the fuel, ${\dot{m}}_{f}^{"}$ (kg/m^2^ s) is the mass flux from the fuel surface, χ is the burning efficiency (≤1) and $∆H_{c}$ (J/kg) is the heat of combustion of the involved fuel. With only a few exceptions, e.g., solid phase oxidation of char coal, combustion takes place in the gas phase (flame) where the pyrolysis products released from the fuel react with entrained oxygen. The mass flux may be expressed by:

(3) $${\dot{m}}_{f}^{"}=\frac{{\dot{Q}}_{F}^{"}+{\dot{Q}}_{E}^{"}-{\dot{Q}}_{L}^{"}}{L_{V}}\;(\mathrm{kg}/m^{2}\;s)$$

where ${\dot{Q}}_{F}^{"}$ (W/m^2^) is the heat flux supplied by the flame, ${\dot{Q}}_{E}^{"}$ (W/m^2^) is the heat flux from any external heat source (such as distant flames, hot smoke layers or hot objects), ${\dot{Q}}_{L}^{"}$ (W/m^2^) is the heat losses from the surface and $L_{V}$ (J/kg) is the latent heat of pyrolysis, including contained water evaporation. Fire spread increasing the involved fuel surface area may in general be caused by direct flame contact (convection), thermal radiation and by airborne glowing embers and firebrands.

### 2.3. Compartment Fire Development

In a developing compartment fire, the smoke layer temperature increases. The heat flux from the smoke layer (${\dot{Q}}_{E}^{"}$) is proportional to its absolute temperature to the 4th power, i.e., it increases dramatically with smoke layer temperature [11]. When the smoke layer temperature approaches 550−600 °C, the heat flux levels are so high that the fire likely passes through a stage of sudden increase in severity called flashover. Post flashover, the hot smoke and flame layer temperatures are typically around 1000 °C and all combustible surfaces are involved in the fire. The mass flow of fuel then usually exceeds the amount being combusted by the air flow into the compartment. The excess fuel burns as flames out of the ventilation openings giving potential spread of the fire to the facades and to neighbor structures. The drier the combustibles are, the faster the fire spreads [26] and the earlier a flashover may be expected [27]. The Fuel Moisture Content (FMC) is therefore of vital importance for fire development and conflagration risk.

### 2.4. Wildfire Development

Wildfires are also governed by the principal Equations (1)−(3). Rather than thermal radiation, it has recently been documented that convective flame heat transfer is the most important near-field fire spread mechanism [28]. Wind is a central factor assisting this heat transfer. As the fire grows, the fire spread may also be assisted by, and governed by airborne transport of glowing embers and firebrands over very long distances, i.e., kilometers [29]. The drier the wildland fuel, the easier it ignites and the more intensely it burns. When dried beyond a certain level prior to a fire, the soil may also be involved in the combustion process. Drying of wildland fuel may be quite fast, also in subzero temperatures and when dead, the fuel usually dries quickly as no live bark layer limits the drying processes [7]. Dieback of potential wildland fuel may be caused by subzero temperature drying (desiccation), such as experienced in the Calluna stands in Norway succeeding the snow-free and cold January and February 2014 [7], or biological attacks, such as the Mountain Pine Beetle (MPB) infestation of the forests of British Columbia, Canada [19]. The drying of wildland fuel (and the soil) is therefore of vital importance for wildland fire risk.

### 2.5. Influence of Fuel Moisture Content (FMC)

Reduced Fuel Moisture Content (FMC) changes all parameters in Equations (2) and (3) for the worse regarding the fire development [8]. The lower latent heat of vaporization ($L_{V}$) is the main contributor. Drying prior to ignition therefore exerts a major influence on the characteristics of wildland fires as well as compartment fires. Understanding the drying processes is therefore necessary in order to understand fire development and fire severity in dry conditions.

## 3. Air Relative Humidity (RH) and Drying Processes

**Figure 1:**
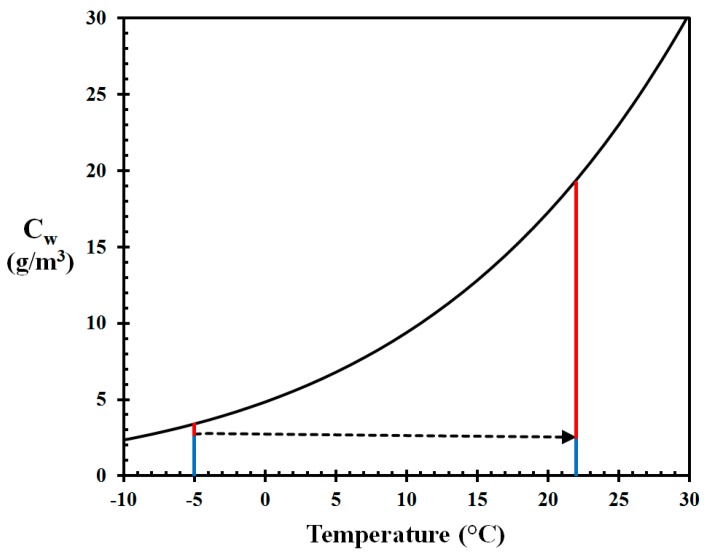
Saturation concentration of water (in air) [30] as a function of temperature (Air at −5 °C and 80% RH heated to 22 °C giving 13% RH indoors). The calculation procedure is given by Log [8].

The vapor pressure of water is a function of temperature. The water saturation concentration in air at atmospheric pressure can be calculated based on the work by Tetens [30], the molar mass of water and the molar gas constant, and takes the form shown in Figure 1. The air usually contains less water than this, and the relative fraction is called the air Relative Humidity (RH). The saturation concentration increases with temperature, i.e., more water can be in the vapor phase. Additionally, when heating cold air to indoor temperatures, it is depleted some in humidity concentrations due to the expansion of gases with temperature. When heated, the air therefore becomes dry, i.e., low Relative Humidity (RH). As an example, outdoor air at −5 °C and 80% RH brought indoor and heated to 22 °C theoretically results in 13% RH indoors, as indicated in Figure 1. Moisture supply from activities like dishwashing, people and pets breathing, pot plants etc. do increase the indoor RH slightly, but in cold climates the indoor air still usually becomes very dry due to the normal air exchange rate. This is confirmed in several studies in Nordic countries, e.g., by Kalamees et al. [31].

Due to the entropy gain involved in the phase change to water vapor, there is a very strong driving force for the free, and also the adsorbed (hydrogen bonded), water evaporation [32], i.e., the drying process. Already in the 1950s, Pirsko and Fons [33] showed that the frequency of urban building fires was correlated with dew point temperature in the winter for selected areas studied in the USA, i.e., lower ambient dew point resulted in higher fire frequency. In countries with cold climates building fires are still more common during winter [34]. High electricity consumption, dangerous heating sources such as kerosene heaters, fireplaces and wood stoves as well as increased time spent indoors increase the fire ignition frequency [35,36]. However, the increased fire frequency may not tell the complete story. The intensity of a fire, e.g., due to low Fuel Moisture Content (FMC), is also important [37].

**Figure 2:**
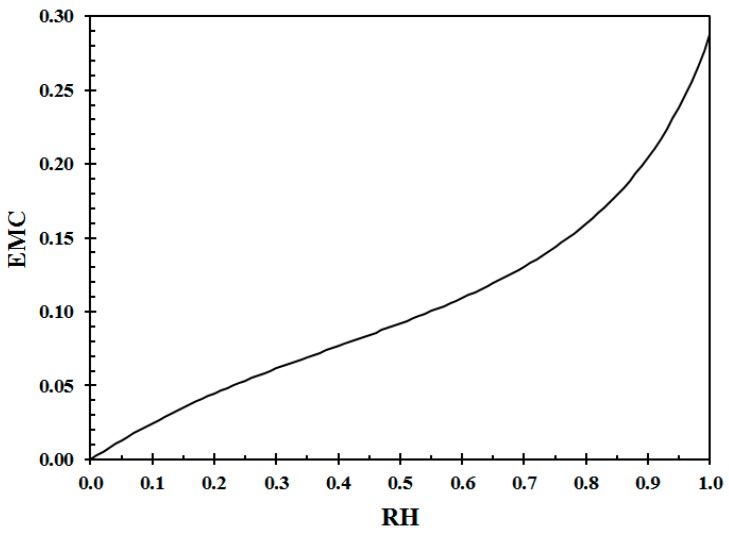
Wood Equilibrium Moisture Content (EMC) at 22 °C (Calculation procedure given in [37]).

Wood and other cellulosic products are hygroscopic (hydrogen bonds). Live plants may contain substantial amounts of water, i.e., >100% relative to being oven dried 48 h at 105 °C [7]. Dead wood, when not soaked contains less than 30 wt % (weight %) water to the dry weight basis. The Equilibrium Moisture Content (EMC) is a function of the air Relative Humidity (RH) shown in Figure 2. There may be some hysteresis [38], but in principle, wood drier or wetter than the actual EMC, will approach the EMC value by absorbing or releasing humidity (drying).

The drying process may be described by Fick’s law:

(4) $${\dot{m}}^{"}=-D_{w}\cdot \mathrm{dC}/\mathrm{dx}\;(\mathrm{kg}/m^{2}\;s)$$

where ${\dot{m}}^{"}$ (kg/m^2^ s) is the mass flux of water, $D_{w}$ (m^2^/s) is the diffusion coefficient of water, C (kg/m^3^) is the water concentration and x (m) is the distance, i.e., dC/dx (kg/m^3^ m) is the concen­tration gradient. The diffusion coefficient is increasing only slightly with temperature. Since the water vapor pressure increases nearly exponentially with temperature, the driving force of the drying process, illustrated by the length of the red vertical lines in Figure 1, is much greater indoors (at 22 °C) than outdoors (at −5 °C). The indoor air therefore dries the interior (wood, upholstery, cloths, etc.) towards the corresponding EMC-value.

It can be shown mathematically, or by experiments, that a 10 mm thick wood panel suddenly exposed to, and kept exposed to, dry air on one side reaches close to the new EMC conditions within 4–5 days at 22 °C [37]. Unless wetted to beyond the fiber saturation point, upholstery and fabrics dry significantly faster to the new EMC condition, i.e., usually within an hour or two. Exposed to similar relative humidity air at slightly subzero temperatures outdoors, wood dries at about 1/5 of the drying rate at indoor conditions, but it still dries. Shrubs with much dead biomass, twigs and thin branches, such as old heather (*Calluna vulgaris* L.), gorse (*Ulex europaeus* L.) and similar stands, which are abundant in certain areas, and represent thin objects, may dry from rain wet to very dry within a day or two in subzero temperatures [7].

### 3.1. Cold Climate Fire Risk

The first cold climate fire that caught the authors’ attention was the Opémiska Community Hall fire that occurred at 01:30, 1 January 1980, in Chapais (Quebec, Canada), which got international media coverage. Tragedy struck when a fire took hold in tinder-dry decorative spruce or fir boughs in the Community Hall, where about 350 persons had gathered for the New Year celebrations [39]. 48 people lost their lives in the fire, and 50 were injured and rushed to the Chibougamau hospital. This was the worst fire to occur in Quebec for more than 40 years. The next cold climate fire that caught attention was the 1994 New Year eve fire at the Switel Hotel in Antwerp, Belgium [40]. Four hundred and fifty people were seated in the banqueting room adjacent to a smaller entrance room, where two Christmas trees caught fire. Due to the lower ceiling height, the fire almost instantly resulted in a flashover. The short (apparently less than 1 min duration), but very severe fire resulted in significant personal and material losses, i.e., 12 people died within a couple of days and more than 140 people were injured. CFD modelling was used to explain the surprising severity of the fire and the health impacts on the people exposed [41]. Grouping these two extreme fires in the boxes “decoration fires” or “Christmas tree fires” may result in the fundamental causes of these fires being overlooked. The previously mentioned indoor low Relative Humidity (RH) and thereby strong drying potential is together with the thin fuel dimensions, the main reasons for fir and pine decorations, wreaths and Christmas trees representing severe fire risks in winter time.

A recent study of fires in Australia showed that there was a higher Odds Ratio (OR) for dying in a home fire during the winter time than during the warmer seasons [42]. During cold months, and given a fire, the OR for death in a fire was 1.9 times higher than survival. In their work, this was not further explained, but it certainly is an indication of more severe fire development in colder weather, in line with the previously described drying potential. It also confirms the experiences from the 1980 Opémiska Community Hall fire in Chapais, Quebec [39], and the 1994 New Year eve fire at the Switel Hotel in Antwerp [40,41].

### 3.2. Fire Risk and Cold Climate Changes

Climate changes are usually manifested as increased temperatures, changed precipitation patterns, windier weather and declining snow pack [43]. Extreme cold events are, however, expected to continue and possibly increase in certain northern regions [44,45].

Strong winds result in a higher number of indoor Air Changes per Hour (ACH) in wooden structures, diluting the previously mentioned indoor moisture supply [37]. Stronger winds also increase the risk of fire spread to other buildings by direct flame contact or by airborne firebrands [3,8].

One may argue that there have always been very dry periods during winters in Arctic areas. In Norway, the locals benefitted from this dry weather by drying stockfish for the international market, etc. However, global warming results in the wildland being exposed to warmer weather, i.e., faster drying, and more exposure to drying due to declining snow pack. When exposed to adiabatically heated (dry) subzero temperature air, without the protective snow layer, winter time wildfires may become an increasing challenge [16].

## 4. Health Impacts of Four Recent Subzero Temperatures Fires

### 4.1. The Lærdalsøyri Fire, Norway, 18 January 2014

In contrast to the windy and very wet December 2013, during January 2014 the weather in large parts of South-Western Norway was cold (subzero), dry and windy. Air from the central Norwegian mountain regions was compressed as a result of higher pressure at sea level and thereby adiabatically heated when descending towards sea level in the easterly gradient winds. This resulted in slightly subzero temperatures and low relative humidity (RH) drying the outdoor wooden materials [8]. Heating this air to indoor conditions at e.g., 22 °C resulted in very low indoor Relative Humidity (RH). 18 January, when a fire started in a private home, external and especially internal wooden materials were very susceptible to ignition and severe burning. The fire developed extremely fast in the home of the fire origin, spread to neighboring homes and threatened the whole village, including the Old Lærdalsøyri cultural heritage site. Destroying 40 structures, including 15 private homes and four historic buildings [8], it was the largest peacetime fire in Norway since 1923 when 250 structures were lost at Hemnesberget [1]. The fire area was about 500 m × 200 m. Spotting ignition by firebrands was identified as the most important fire spread mechanism [46]. This fire came as a surprise to the local firefighters, as well as the national fire authorities, i.e., a typical Black Swan event [47]. This risk was known to some [8] and may therefore be characterized as an “unknown known” Black Swan event [48].

About 680 persons were evacuated and fortunately, this conflagration did not result in any fatalities. Some inhabitants did, however, refuse evacuation orders, hid from the police forces and continued firefighting with garden hoses, cans and buckets, exposing themselves to the dangers of the fire. Four hundred and forty six persons were checked in at the local hospital and 270 were hospitalized for shorter or longer periods, most of them due to smoke exposure and burns from embers and firebrands [49]. About 70 persons lost all their possessions.

The direct smoke exposure and hospitalization is generally recognized by the society, as well as the direct loss of property. Such a dramatic and frightening conflagration also has considerable psychological effects, to which each person may react differently. All are marked for life, and some may not be able to resume regular work for years. There was also surprisingly low willingness by the Norwegian central authorities to support the small Lærdal community to cope with this out of proportion major incident. There was no crisis fund to help the society regain balance afterwards. This added to the stress of the locals and the community administration.

### 4.2. The Résidence du Havre Nursing Home Fire, Quebec, 23 January 2014

Five days later, a far more devastating cold climate fire took place at Résidence du Havre nursing home (senior home) in L’Isle-Verte, Quebec, Canada, 23 January 2014, resulting in 32 fatalities and 15 injured persons [9]. The nursing home was built in two phases. Phase I, a unit for residential use, was built in 1997 before sprinkler requirements were enforced while Phase II, a supervised residence, was built in 2003, under rules requiring an automatic sprinkler system. In the case of fire, the emergency procedure was to direct the inhabitants of Phase I to a safe area, i.e., to the Phase II.

Strong winds were mentioned by several of the interview subjects in the coroner’s report [9]. It is evident from photos from the scene of the fire, and reports of fire victims covered by a foot of ice, that it was very cold during the fire. According to Roslin [50] “It was one of the coldest days of an unusually nasty winter, even for L’Isle Verte. A bracing northern wind blew all day out of the snowy Charlevoix Mountains. It tore across the frozen mouth of the St. Lawrence River, 25 km wide at this point, and blasted the village of 1400 nestled on the river’s south shore, six hours northeast of Montreal.” 

Data from Riviere-Du-Loup weather station (http://climate.weather.gc.ca) at Ile Rouge, 10 nautical miles upwind of L’Isle Verte, show that during the last three days before the fire, the wind strength was about 10–17 m/s from North West and the temperatures were in the range −22 °C to −15 °C. The relative humidity (RH) was around 80–90%. Heating air at −15 °C and 90% RH to 22 °C theoretically results in 6.5% RH indoors, i.e., well below the theoretically calculated 11% RH prior to the Lærdalsøyri fire five days earlier [8]. Indoor activities increase the relative humidity some. The interior potential combustible cellulose based fuel must, however, have been very dry and susceptible to intense combustion prior to ignition.

The human losses in Résidence du Havre nursing home fire are beyond proportions. An analysis of the health issues is therefore not done as it is understood by all readers that this was a most tragic event with huge health impacts for all victims, relatives, firefighters, local community administration, neighbors, etc.

### 4.3. The Flatanger Wildland Fire, Norway, 28 January 2014

In Norway the cold dry weather continued throughout January 2014. There was no snow cover in the coastal scarcely inhabited lowlands in Nord Trøndelag. The dry and windy weather desiccated (freeze dried) vegetation and soil. In large areas along the coast, such as Flatanger, regular maintenance of heather by frequent controlled mosaic burning had previously been performed for optimizing herbivore production, i.e., mainly sheep grazing. After World War II, this landscape use was gradually abandoned. This allowed the area to build up significant amounts of elevated biomass, especially in the form of heather (*Calluna vulgaris* L.) with much dead elevated lower canopy and litter, i.e., biomass very susceptible to drying and potential subsequent combustion.

27 January 2014, 22:00 local time, strong winds (20 m/s, peaking 28 m/s) resulted in short circuit sparks from suspended electrical power cables igniting dry and snow-free grass/heather [49]. The strong wind resulted in fast fire spread during the long dark Nordic winter night, just 210 km south of the Arctic Circle. Fortunately, all inhabitants were evacuated before the fire became life-threatening. The firefighters struggled to control the fire. They partly had to lie down as the strong winds blew them over [49]. The terrain was rugged, there was very limited access to the area and the firefighting equipment quickly froze. After burning 15 km^2^ of *Calluna* dominated Atlantic heathlands and destroying 63 wooden structures, among these 23 villas and vacation homes, the fire finally terminated itself at the North Sea. With respect to the number of lost structures, this wildland urban interface (WUI) fire was even larger than the Lærdalsøyri fire, 10 days earlier. Due to early evacuation and care during firefighting no persons were hospitalized. But still, returning back to your home to find nothing but ash, broken cups, plates and twisted kitchenware takes its toll psychologically. To alleviate some of this pain, members of The Norwegian Civil Defense emergency and rescue departments joined the owners on their first visit to their previous homes.

Norway uses civil, and may use military helicopters for fighting wildfires. The sound reason for firefighting helicopters rather than airplanes is based on the abundancy of small lakes serving for helicopter basket refilling. In the strong winds, the helicopters could, however, not be used before the 3rd day of the fire. In the subzero temperatures, the lakes were frozen making water basket refilling a challenge. The fire was not deep seated, i.e., it did only to a minor extent involve soil combustion. The seed banks were not destroyed and the recovery has been fortunately fast [7].

### 4.4. The September 2007 North Face Tundra Fire, Alaska

A significant increasing number of fires have been recorded at the North Western Alaskan tundra during the period 1950–2006 [51]. A late September 2007 North face megafire burned an area of 1000 km^2^ at 69° N. It is the largest fire on record in Alaska north of the Arctic Circle. Joly et al. [51] considered it remarkable that it burnt on the tundra during late September, when there was already ice on the small lakes. This fire made local residents concerned about Arctic tundra fires becoming an issue in the future.

In tundra ecosystems, wildfires reduce the abundance of lichens for decades. The long time needed for developing the late-succession fruticose lichens representing the primary caribou forage makes the caribou avoid previously burned tundra habitats in mid-winter for up to 55 years [6]. This is not much less than the 76 years needed for full reestablishment of perennial plant cover in the American Southwest Mojave and Sonoran deserts [52]. Given the likely climate warming and changing fire patterns, it is predicted that the Central Arctic caribou herd wintering primarily in the arctic tundra can expect 11% reduction in lichen-producing winter habitats [53].

In a long time perspective, the loss of jobs related to caribou production and restructuring of the life style does not come without its psychological challenges. This is also observed for wildfires in the Mediterranean area [54]. And, as concluded by several researchers, e.g., Ostry et al. [19], the rural population is especially vulnerable to such changes. The potential of future deep seated fires in Arctic areas is also a concern. The possible change of arctic areas from mega scale carbon storage areas to mega scale CO_2_ releasers, increasing the global CO_2_ concentration and accelerating the global warming, is a major general concern. This late September 2007 North face megafire can be a warning sign for future health concerns in the Arctic areas.

## 5. Risk Implications

Both the official investigations after the Lærdalsøyri, L’Isle-Verte, and Flatanger fires focused on training of volunteer and part time firefighters and on regrouping and unifying fire departments to make stronger units, especially in rural and distant areas [9,49]. Trained firefighters from larger fire stations would probably do an even better job. Would this, however, make a difference if they regardless arrive too late in the case of very fast developing fire? This question must be seen in relation to the analysis by Holte et al. [55] who concluded that the emergency responders in the Lærdalsøyri fire and the Flatanger fire did indeed act within the prevailing rules and regulations. In the mentioned investigation reports there are no references to indoor drying of wooden materials. If asking individual officers and decision makers, they would probably agree that very dry wood burns more intensely than more humid wood. They may perhaps also know that it generally becomes very dry indoors during cold winter weather. Combining these two facts and relate them to the fire risk may, however, not be trivial. These fire risk impacts are therefore briefly outlined.

**Figure 3:**
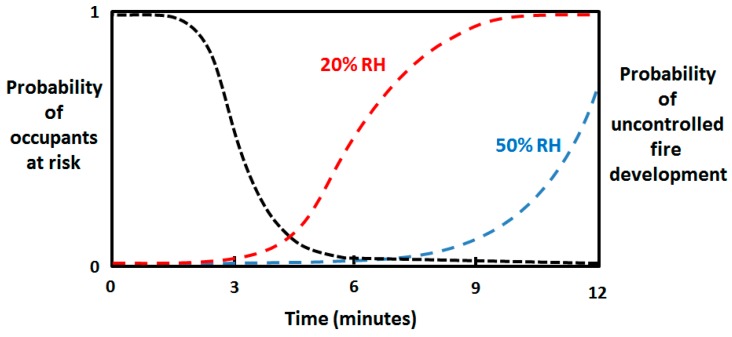
Sketch of people at risk (**- - -**) versus fire development in normal (- - - 50% RH) and dry (- - - 20% RH) conditions.

The time needed for a 2.5 kW fire to develop to flashover in 1/4 scale wooden pine test rooms of varying Fuel Moisture Content (FMC) was recently studied by Kraaijeveld et al. [27]. The time to flashover was reduced from 8 min for wood at 9.3 wt % FMC to only 4 minutes at 4.5 wt % FMC. At room temperature, this respectively corresponds to about 50% RH and 20% RH [37]. Faster development of a fire in a detached home, with (in Norway) mandatory smoke alarm systems installed, may not represent a significantly increased risk to mobile inhabitants. They will most likely still have sufficient time margins for evacuation, as indicated in Figure 3. This is in line with the general belief that the occupants need to self-evacuate long before the fire service arrives [56]. For people with impaired mobility it may, however, be the difference between being safely rescued or exposure to the direct dangers of the fire. In a large construction, where evacuation takes more time, or where the inhabitants experience impaired mobility, early flashover due to dry indoor conditions may result in catastrophic consequences, such as the Résidence du Havre nursing home fire [9]. The cold climate fire risk is therefore in particular a threat to vulnerable groups. This is also emphasized by other researchers studying health impacts of fires [19,20].

If additionally there is much wind, early flashover may result in very fast fire spread to close and distant structures representing a huge challenge for the firefighters [29]. Indoor relative humidity of inhabited (heated) wooden structures may therefore represent an important indicator regarding the fast development and severity of a potential building fire [37]. Such a technical telltale may be important for contingency planning, resource allocations [57] and warnings to the public about increased fire risk.

When studying the relationships between fire service response time and fire outcomes in New Zealand, Challands [56] discovered that the fraction of fires considered large, i.e., over 30 m^2^, was linearly related to the response time x (minutes). The linear relationship for 27,500 structural fires gave y = 0.023 min^−1^·x + 0.043, i.e., 2.3% more fires had grown beyond 30 m^2^ for each additional minute response time. For very dry wood fires, the relationship may be similar, but probably with a steeper slope as the fires reach flashover earlier.

To understand the difference between e.g., 10 min and 6 min to flashover (and a fully engulfing home fire) for the emergency response system, we may investigate a time line for the fire fighters actions. We then simply compare 10 min available for the firefighters in the normal condition to 6 min for cold climate dry wood condition. For simplicity, we may assume that the fires are discovered instantly by a fire alarm sounding to warn the inhabitants. According to Averill et al. [58] it takes about 60 s to verify a fire alarm, evaluate the situation and eventually inform the 911 central. A 60 s processing time at the 911 alarm central and a turnout time of 80 s can be expected for manned fire stations [59]. When at site, 40 s may be added to get oriented, roll out fire hoses and start fighting the fire, i.e., Water on Fire (WOF). This adds up to 4 min. For simplicity, we further assume that fire vehicles drive at 60 km/h and that all roads go radially out from the fire station, no traffic jams, etc. In the normal case this leaves 10 − 4 = 6 min for driving before it is too late to control the fire, i.e., the range is 6 km. In the dry wood case, they may drive for 6 − 4 = 2 min, i.e., range 2 km. The area coverages (π·r^2^) turns out to be 113.1 km^2^ and 12.6 km^2^, respectively, i.e., the area coverage in the dry condition is only 1/9th of the area coverage in the normal case. Part-time on-call firefighters will need additional time to get to the fire station, making this risk picture even worse. This was indeed the case in the Lærdalsøyri [8] fire, the L’Isle-Verte [9] fire and the Flatanger fire [7]. During winter time in cold climate areas, snow and icy roads may also add to the time needed for the firefighters to get WOF.

**Figure 4:**
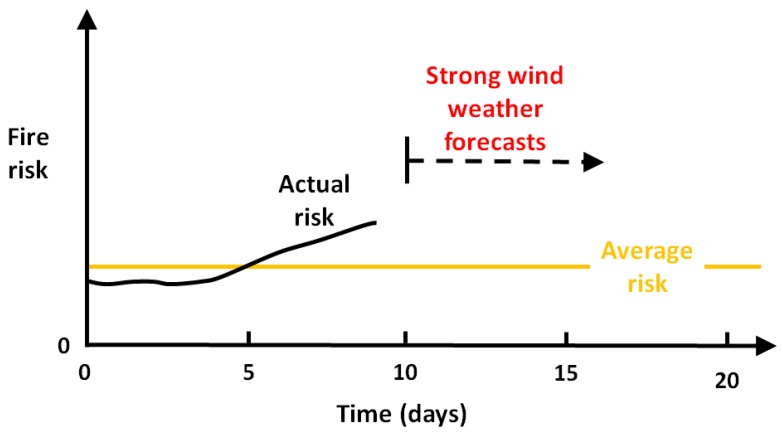
Sketch of indoor fire risk development at day 9 during a cold snap with adverse wind forecast for the next days.

One may question whether risk peaks related to cold climate fires are sufficiently understood. An example of the average risk and an imagined actual risk is illustrated in Figure 4. In the sketch, it is indicated that the risk was below the average during the first 5 days. Then, the weather became cold and dry for the rest of the period shown on the figure. At day 9, the wood is quite dry, i.e., some higher risk, as indicated also in Figure 4. Then, from day 10 there is a weather forecast predicting very strong winds for about one week. From day 10 to day 17 very high risk peaks can be expected making the average risk irrelevant in this period. The fire brigade units are, however, dimensioned in accordance to this average risk (with perhaps 30% safety margin?). People are confident that the fire fighters will manage to handle the fire. Then, when a fire disaster strikes at e.g., day 14 everybody seems surprised. The authors fear that severe risk peaks may be overlooked based on the yearly risk average. However, at day 9 in the example Figure 4, if the windy days’ risk is drawn and visualized, a better understanding of the future risk may be obtained.

## 6. Potential Mitigating Measures

### 6.1. Establishing the Mental Risk Picture

As there seems to be a lack of understanding/acknowledgement of the increased risk regarding cold climate fires, the knowledge of the underlying dry wood fire dynamics must be improved to prevent these fires, or to extinguish them early [8]. In a risk perspective, reducing the number of fires must be priority number one in order to reduce the health impacts. The second priority must be to extinguish the fire, i.e., get water on the fire before it becomes too severe. This could be achieved by automatic sprinklers, water mist systems, etc. or by early interference by capable persons using a fire hose. Further research on how to improve the mental risk picture is encouraged.

### 6.2. Table Top Training

Training firefighting skills and incident command co-ordination may be done using physical or virtual simulators as a cost-effective and safe alternative to practice with real scale fires. The recent development in video games and new consumer-level physical video interfaces is promising. Simulations and Serious Games (SSG), games whose primary purpose is education and training, may soon become a proper training tool [60]. As an example, practicing a few scenarios with normal case fire development, including the mentioned time delays needed to validate the alarms, mobilize, drive to the fire scene, organize fire hoses and start fighting the fire, may make the participants familiar with normal case fire development. The next scenario could then be a winter month (very dry indoor conditions) fire which develops twice as fast. The pure shock of (always?) arriving too late to control the fire will probably make the students think quite seriously about why this happened. Using real cases is suggested also by Heldal et al. [61] to make the most out of SSG practices. Learning from contingency exercises and severe incidents in other sectors may also be worthwhile [62,63]. Further research on optimizing emergency training regarding cold climate fire risk is encouraged.

### 6.3. Practical Physics and Enclosure Fire Dynamics Demonstrations

**Figure 5:**
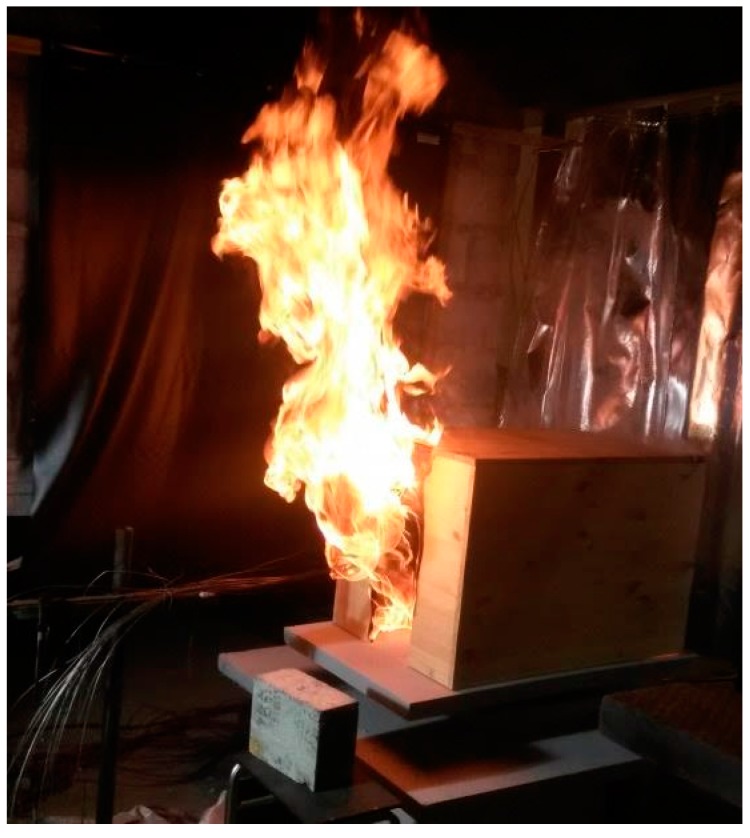
Small scale (1/4) pine wood (5.1 wt % FMC) compartment fire just post flashover through an open doorway. (Photo by T. Log.)

Weighing the same wood sample exposed to dry and humid conditions may convince the audience that the dry wood contains significantly less water than more humid wood. Simple consumer market humidifiers and dehumidifiers placed in small plastic tents may be used as low cost wood conditioners. Then, demonstrating a ¼ scale normal case FMC value wooden compartment fire and a similar dry wood fire will clearly show faster dry wood fire development [64], see Figure 5. Further research on practical physics regarding drying and combustion is encouraged.

### 6.4. Practical Physics and Wildfire Dynamics Demonstrations

Similar practical demonstrations may also be done using small scale models illustrating wildfire risk. Conditioned mockups of humid and dry 3–4 mm diameter wood cylinders representing a wildfire fuel of different FMC values may simulate the characteristics of wildfire fuels. Igniting a 2 m tall dry juniper (*Juniperus communis* L.), an abundant resinous “weed” in successional North Atlantic coastal heathlands, would serve the spectators with an unforgettable “don’t do this at home” fire. Further research on practical physics regarding drying and wildland fuel combustion is encouraged.

### 6.5. Learning from Other Sectors

Some climate changes are easily observable, such as cold spells, declining snowpack, strong wind forecasts, etc. The problem may be to recognize the local impacts and significant risk peaks. Maybe a modified HAZID (hazard identification) study adopted from the industry, with proper check lists, can be a step forward in identifying changing fire risks. Then, some sort of hazard and operability (HAZOP) analysis, where each identified “node” is analyzed for changes like more, less, lower, higher, etc. could be useful for understanding the variability in the identified risks. The As Low As is Reasonable Practicable (ALARP) principle, originating from the UK mining industry, and adopted as a regulatory requirement by the health and safety authorities in many countries for the oil & gas industry [65], may also be a guidance. In the ALARP spirit one has to demonstrate that each possible Risk Reducing Measure (RRM) identified must be grossly disproportionate in cost or effort compared to the associated risk reduction achieved in order not to be implemented. Such approaches may help preventing the pitfall of “the yearly average risk is OK’-thinking. Further research on applying industrial hazard and risk analysis methods on civil sector fire risk is encouraged.

### 6.6. Relocate Emergency Responders According to the Risk?

Relocation of firefighters and fire trucks has been suggested for Santiago, Chile, to better handle the temporal and spatial variation in fire risk, i.e., WUI fires in the outskirts of the city in the summer (October to March) and wooden structure fires in the old city center in the winter, i.e., April to September [57]. Relocation of firefighters closer to high risk areas during identified risk peaks may reduce the health impacts of such fires. Further developing such ideas is encouraged.

### 6.7. Automatic Fire Suppression Systems

Installing automatic sprinklers, especially where people with limited mobility reside, is currently an issue in Canada. Price has always been claimed to be a serious obstacle. Functional measures with a better cost/benefit ratio, such as flashover prevention by water mist, can be considered [66]. Depleting the oxygen concentrations, while still keeping the respiration in balance, i.e., using inert gasses with added CO_2_ for enhanced respiration, could also be considered if proven safe for the groups at risk [67]. Further research on alternative low cost fire suppression methods is encouraged.

### 6.8. Wild Fire Risk Mitigation

Other preventive measures may work better for mitigating cold climate dry weather wildfire risk. As an example, at Flatanger, regular controlled burning would reduce the fire risk in dedicated buffer zones and also be beneficial for the heather [68] as this plant is adapted to anthropogenic fire regimes [69]. Planned burning may be difficult in large areas. It can, however, be very beneficial in strategic locations, e.g., between lakes and other natural fire breaks, especially in close proximity to hamlets at risk. This will also keep the growth of the highly flammable resinous foliage juniper (*Juniperus communis* L.), burning like torches in the Flatanger fire [70], under control in the buffer zones. Recognizing an increasing risk by keeping an eye on the weather development and especially the predicted RH values will help prepare firefighters for any increased WUI risk. Proper early action may reduce the consequences significantly. This also holds for potential megafires in Arctic areas. The huge distances and limited access roads do, however, make it extremely difficult to fight such remote fires. On the other hand, if detected early (by e.g., remote satellite IR cameras), there might be a chance of controlling these fires. Further research on using prescribed burning to develop efficient fire barriers is encouraged.

### 6.9. Mitigating Children Fire Distress

To relieve the distress among children that have lost their home, the Swedes invite these children to the fire station, talk to them and show them fire vehicles and other firefighting equipment [71]. The idea is to let the children have other stories to tell their peers about fires other than their losses only. Preparing the firefighters for such tasks may help the children coping better with traumatic stress reactions.

## 7. Discussion

The general dangers of the fires to humans, i.e., civilians and firefighters, as well as the impact of megafires on the environment have been briefly presented. Four subzero temperature fires have been analyzed in more detail. These fires span the range from fires in low density wooden town area (Lærdal, Norway), a devastating nursing home fire (L’Isle Verte, Canada), a Wildland Urban Interface fire (Flatanger, Norway) and a wildland megafire (North Face, Alaska). It is demonstrated that the drying of wooden products had a central role in all these fires. The health impacts range from indirect issues such as loss of homes and jobs and mass evacuation to hospitalization and 32 lives lost in a single fire. A span in fires like this has both limitations and strengths. More knowledge could have been revealed by studying each type of fire in more detail. However, studying this range of fires may make it easier to recognize that the drying of wood and other cellulosic materials entails a fire threat that appears to have slipped below the radar. It is especially challenging to the most vulnerable people. In combination with strong winds, dry cellulosic combustibles may result in conflagrations due to the fast and long distance fire spread, both in towns as well as in the wildlands and wildland urban interface.

Methods to improve the risk understanding are suggested. This is by no way a complete list of topics but may serve as inspiration for research reducing the impact of climate changes on the cold climate fire health risks. It is the opinion of the authors that when the risk is understood, fire officers and firefighters may be trained to identify risks and take mitigating actions fit for the situation under consideration. When understanding the risk they would at least be better prepared to handle an incident.

One may question whether the four fires discussed are a result of climate changes. With respect to the Résidence du Havre nursing home (senior home) fire, it has “always” been cold in Quebec in January. The same holds for Lærdalsøyri, Norway. However, the combination of strong winds and low temperatures/dry weather is quite unusual, at least in Lærdal. In Flatanger, the combination of no snow, subzero temperatures and strong desiccating winds is highly unusual, and declining snowpack is a certain sign of climate changes [19]. The increasing summer temperatures in the Alaskan North Slope are also a climate change signature. The authors believe, but cannot prove, that climate changes played a role in all these fires. As the frequency of cold spells is predicted to increase in northern regions due to climate change [44,45], health issues due to cold climate fires risk can be expected to increase. It is therefore important to be aware of this development and carefully identify the increased risk of extreme fire events. Otherwise, this can become a growing public health issue.

Mitigating structural cold climate fire risk peaks may include campaigns for reducing the number of fires, relocation of firefighting resources for sufficiently early water on fire, firebrand intrusion barriers limiting spread into new structures, etc. Mitigating measures for WUI fires may include prescribed and controlled burning of fire buffer areas in the terrain close to people at risk. Mitigating the effects of Arctic mega fires is probably best achieved by reducing global warming, to which these fires may contribute. As the drying can be predicted based on weather forecasts, periods of increased risk may be monitored and even forecasted. This is a very important first step in future fire prevention and mitigation of the potential health impacts of the possibly increasing number of cold climate fires.

## 8. Conclusions

The health impacts of cold climate fires are significant, as demonstrated by the four subzero temperatures fires discussed in the present work: a wooden village fire, a single wood structure fire, a wildland urban interface (WUI) fire and a huge wildland fire. These four fires resulted in 32 fatalities, 385 persons hospitalized for shorter or longer periods, 104 structures lost and 1015 km^2^ wildland burned north of, and just south of, the Arctic Circle. Medical effects of smoke and heat exposure and psychological effects related to loss of relatives and loss of possessions are severe. It is shown that a combination of environmental factors such as subzero temperature dry weather, strong winds, declining snowpack alone, or in combination with changing agricultural activities, may involve threats to people not previously anticipated. Such multi-discipline factor combinations may not be easily identified as risk contributors. The effects of indoor dryness and risk implications regarding fire development involving dry wood are discussed. Methods for risk identification and general training in understanding contributing risk factors are suggested for reducing the impacts of a possibly increasing number of such fires in the near future.
